# Can the natural resource balance sheet system promote ecological civilization? --Empirical evidence from panel data of prefecture-level cities in China

**DOI:** 10.1016/j.heliyon.2024.e33463

**Published:** 2024-06-27

**Authors:** Le Zhu, Yichuan Wang, Shengchuan Guo

**Affiliations:** a School of Accounting, Shandong University of Finance and Economics, Jinan, 250000, China; b Department of Economics, University at Albany, SUNY, Albany, NY, 12222, United States

**Keywords:** The natural resources balance sheet, Ecological civilization construction, Natural resource and environmental information, Government governance efficiency

## Abstract

As a crucial carrier of natural resource and environmental information, the natural resources balance sheet is important in enhancing ecological governance capabilities and advancing ecological civilization construction. Utilizing panel data from Chinese prefecture cities spanning 2012 to 2018, we employ a Difference-in-Differences (DID) model to examine the policy effects of the natural resources balance sheet system. The findings indicate that compiling the natural resources balance sheet significantly promotes local ecological civilization construction, and local government environmental governance efficiency partially mediates this relationship. Further analysis indicates that the outgoing audit of natural resources and public environmental concern positively moderates the relationship between the two. Drawing on the principal-agent theory, we provide in-depth analysis and elucidation of the mechanisms through which the natural resources balance sheet promotes ecological civilization construction, offering theoretical guidance and empirical data support for strengthening the compilation and application of the natural resources balance sheet.

## 1 Introduction

Ecological civilization construction stands as China's proactive response to the global trend of sustainable development. Undoubtedly, the remarkable growth of the Chinese economy represents a marvel. however, this economic surge has incurred substantial costs, resulting in severe resource depletion, environmental pollution, and ecological degradation. To strike a balance between economic development and environmental protection, China introduced the concept of ecological civilization in 2007 [1]. Ecological civilization involves a developmental philosophy and practice that, within societal progress, advocates for the rational utilization of natural resources, environmental preservation, and the promotion of sustainable economic development [2].

The Chinese government places great emphasis on ecological civilization, implementing a series of policies and institutional measures to construct a comprehensive ecological civilization system. The overarching objective is to comprehensively address ecological and environmental restoration and protection across all regions, fostering the harmonious co-development of the economy and ecology. Nevertheless, despite implementing many practical measures, China grapples with severe issues of resource wastage and environmental pollution. This situation is intricately linked to the long-standing official promotion system that excessively prioritizes GDP growth in China [3]. The fundamental challenge is to quantify and value natural resources and ecology, which makes them impractical as key indicators for evaluating officials' performance. Consequently, local government officials tend to prioritize economic development overly, often neglecting the adverse impacts of such development on resources, the environment, and ecology. Acknowledging this challenge, on November 12, 2013, the Chinese government explicitly proposed the Natural Resource Balance Sheet (NRBS) system [4].

Undoubtedly, information plays a pivotal role in policy formulation and crisis management. Research by Obst and Vardon (2014) indicates that national accounts have become an indispensable tool for decision-makers in central banks, the fiscal and financial sector, and development agencies, facilitating the coordination of economic information in a concise timeframe [5]. Analogous to the multifunctionality of national accounts, the NRBS encapsulates mass information about the resource and environment, delivering structured insights to its users. The NRBS is a management statement that includes a comprehensive set of quantitative tables that account for the quantity and value of natural resources at a specific time. It describes how natural resources are utilized in a specific region during a specific period and evaluates its impact on the ecological environment. It aims to provide decision-making support for scientific and effective management and protection of natural resources by the main body of environmental governance. Additionally, it serves as an information basis for the main body of the supervision and management of resource utilization to strengthen supervision and management [6].

Existing research underscores the practical value of natural resource accounting and the NRBS. Ochuodho et al. (2016) assert the necessity of integrating natural capital into the national accounts system, emphasizing its pivotal role in effective natural resource management and policy analysis [7]. Song et al. (2019) contend that compiling the NRBS can bolster government oversight efficiency and elevate resource management standards [8]. Luo et al. (2019) propose the incorporation of both physical and monetary accounting of atmospheric environmental resources into the NRBS, which not only elucidates government ownership and affordability of natural resources but also mitigates the risks of environmental degradation stemming from unchecked economic growth [9]. Jin et al. (2021) suggest that the forest resource balance sheet can accurately reflect the stock and fluctuations of forest resource assets, offering crucial support for forest resource early warning systems, monitoring efforts, and decision-making processes [10]. While prior studies mainly engage in theoretical discussions on the functions and roles of the NRBS, the effectiveness of the NRBS system in promoting local ecological civilization construction is an important question which remains unanswered and unverified. However, the lack of systematic research has hindered the full realization of the unique value of the NRBS system as an integral component of the ecological civilization system. This paper aims to address this backdrop by analyzing panel data from Chinese prefecture-level cities between 2012 and 2018. We use the DID model to investigate the impact of the NRBS on local ecological civilization construction and provide evidence supporting its role in the process of ecological civilization construction.

The potential research contributions of our paper are as follows: First, it conducts analysis and testing of the impact of the NRBS system on local ecological civilization construction based on principal-agent theory, supporting the crucial role of the ecological civilization system in serving ecological civilization construction. Notably, while prior researches predominantly focus on the theoretical framework of the NRBS and the practical operation of the NRBS system, our research, uniquely contextualized within the Chinese landscape, extends the discourse by empirically scrutinizing its policy influence through a quasi-natural experiment, pioneering empirical inquiry in this domain. Second, our paper identifies the intrinsic mechanism of the NRBS in promoting the construction of local ecological civilization, providing valuable insights for effectively utilizing the NRBS. Third, from a moderating effect perspective, it delves into the outgoing audit of natural resources and public environmental concern on the policy effect of the NRBS system, supplying empirical evidence to optimize policy synergy and establish a multi-dimensional governance system for ecological civilization. Our research not only augments the theoretical understanding on the NRBS but also provides practical guidance for policymakers and stakeholders invested in fostering sustainable development paradigms.

## 2 Institution background

Under China's property rights framework, the State collectively owns natural resources. Natural resources management and utilization adhere to the "unified ownership and hierarchical management" model. This model suggests that the State maintains unified ownership of natural resources and delegates the exercise of ownership to the central government. Given the complex characteristics of natural resources, the central government further delegates management authority to local governments, assigning specific responsibility for resource management to these local entities and their subordinate departments. While this hierarchical management model has successfully developed, utilized, and preserved natural resources, the separation of ownership and management has given rise to a multilayered agency relationship, escalating agency costs. Especially in the context of China's "official promotion tournament" and fiscal decentralization reform, the opacity of the natural resource "property" has intensified opportunistic behavior among local government officials, escalating ethical risks in resource and environmental management and impeding the construction of ecological civilization.

To address the agency problem arising from the lack of environmental information, the Chinese government proposed in 2013 to "explore the compilation of natural resources balance sheet and conduct the outgoing audit of natural resources." In 2015, the General Office of the State Council issued the NRBS Compilation Pilot Program, advocating for integrating the NRBS system into the ecological civilization system. The pilot program designates specific areas, such as Hulunbeier, Inner Mongolia; Huzhou, Zhejiang Province; Loudi, Hunan Province; Chishui, Guizhou Province; and Yan'an, Shanxi Province, for NRBS preparation. The document emphasizes that "the NRBS compiled in the pilot areas should be made public to society," marking the official launch of China's NRBS compilation.

After 2018, the national and provincial levels fully launched the NRBS compilation following the proposed work plan. In 2020, the National Bureau of Statistics (NBS) issued the "Program for NRBS Compilation (for Trial Implementation)," emphasizing the role of the NRBS in providing information and decision-making support for sustainable resource utilization.

Since 2015, some regions in China have actively conducted pilot work on compiling the NRBS, forming models with guidance, demonstration, and replicability. For instance, the Huzhou model in Zhejiang, the Chengde model in Hebei, and the Loudi model in Hunan have accumulated substantial experience in compiling the NRBS. After 2018, based on summarizing the experience of pilot areas, regions have been actively exploring the formulation of trial regulations for the NRBS, creating a situation where various regions are compiling the NRBS.

## 3 Literature review and hypothesis development

### 3.1 The natural resources balance sheet

The "natural resources balance sheet" is a distinctively Chinese concept. It has captured widespread attention within China's theoretical and practical spheres since its inception in 2013. Due to institutional disparities between China and other countries, there has been limited international research on the "natural resources balance sheet." Existing studies have predominantly focused on practical environmental and economic accounting exploration. The System of Environmental-Economic Accounting (SEEA), recognized as one of the most influential macro-accounting systems, quantifies the environmental dimension by incorporating capital stock calculations. The practical exploration of environmental-economic accounting within the SEEA framework offers China a robust experience and reference.

The NRBS is a comprehensive account of the quantity and value of natural resources at a specific time. This statement reflects the utilization status of natural resources and their impact on the ecological environment in a specific region over a defined period [11,12]. The statement encompasses the quantity and value of natural resources owned by equity entities. It details the flow of reporting elements in the region during a specific period and provides information on the physical quantity and quality of natural resource assets, liabilities, and net assets in the region, as well as the value of resource elements [13]. In terms of the structural framework design of the natural resources balance sheet, Y. Chen et al. (2015) assert that, following the traditional balance sheet equation "Assets = Liabilities + Owner's Equity," the statement should include three major elements: natural resources assets, liabilities, and net assets [14]. Given the unique character of natural resource property rights, which are generally managed and used by governments at all levels, compiling the NRBS falls under the purview of governments at all levels and their subordinate departments [15].

The NRBS possesses accounting and management attributes, serving managerial and supervisory. As a critical tool for government monitoring, it furnishes information and systematic support for natural resource management and environmental protection in China [8]. Luo et al. (2019) contend that incorporating the physical and monetary accounting of atmospheric environmental resources into the natural resources balance sheet can enhance environmental quality and support the harmonious development of the economy and the environment [9]. Jin et al. (2021) also argue that a forest resource balance sheet can systematically reflect the contribution of forest resources to the economy, ecology, and society in both physical and value quantities, which improves the performance evaluation and responsibility accountability of entities involved in the management and protection of forest resources, providing crucial information for early warning, monitoring, and decision-making [10]. Examining water resources as a case study, Liu et al. (2022) observe that, since the introduction of policies such as the NRBS, water resource management performance in the Wuhan region has exhibited relative improvements, achieving positive progress in integrated water resources regulation and pollution control [4]. Wilde et al. (2021) find that comparative analyses of resource quality, as well as normalized comparisons with the total consumption of each resource or its availability, have proven valuable in providing insights into the relative use of resources and the risks and vulnerabilities associated with specific resource losses [16].

### 3.2 Hypothesis development

Given the decentralized nature of the Chinese system, local governments are granted significant autonomy in environmental management affairs by the central government. This autonomy allows local governments to leverage information for decision-making related to environmental policies and the development and utilization of natural resources within their jurisdictions. This decentralized system, however, introduces inefficiencies, primarily manifested as "benefit spillover," leading to reduced regional efforts in environmental enforcement and "free-riding" behavior [17]. Moreover, incentivized by economic growth assessments, local government officials may prioritize economic benefits over ecological considerations. These challenges stem from the weakening of incentive and constraint mechanisms and the dysfunction of resource supervision caused by information asymmetry between central and local governments.

The NRBS serves as a crucial repository of information about natural resources and the environment. It plays a pivotal role in addressing information asymmetry within resource and environmental management. On the one hand, the meticulous compilation of the NRBS necessitates a thorough investigation into the fundamental information and stock flow data of a region's natural resources and environment. This process unveils the local natural resources and their transformations, mitigating information asymmetry resulting from agency relationships in resource and environmental management, reinforcing incentive and constraint mechanisms, and regulatory oversight. On the other hand, the compilation of these balance sheets elevates the importance placed by local governments on ecological civilization construction. It prompts local governments to fulfil their agency responsibilities for resources and the environment actively, subsequently enhancing efficiency in developing and utilizing natural resources, environmental protection, and fostering the advancement of ecological civilization.

#### 3.2.1 The natural resources balance sheet and ecological civilization construction

A primary objective of compiling the NRBS is to understand natural resources clearly. In the context of the incentive and constraint mechanisms of resource and environmental management, the NRBS not only accounts for the natural resource assets owned by the local government but also underscores the responsibility for improving the ecological environment and ensuring natural resources for sustainable development. It clarifies the rights and obligations assigned to the region's central resource management body, effectively averting short-sighted behavior in local government resource management. Additionally, the NRBS offers quantitative indicators for the central government to assess and evaluate the local government's fiduciary environmental responsibilities. This enables better integration of environmental protection indicators into the government officials' performance appraisal system, incentivizing local governments and officials to prioritize environmental protection and sustainable development.

For the regulatory mechanism of resource management, the central government can gain comprehensive insights into the resource management situation of local governments through the NRBS. This circumvents excessive investments in monitoring and investigation, reducing the information collection cost of government regulation. Moreover, the NRBS provides key data and indicators that comprehensively reflect the local government's resource utilization status and ecological and environmental protection policy implementation. The aggregation of this information enhances the efficiency and precision of government supervision. Additionally, the NRBS systematically presents information related to local leaders' ecological and environmental responsibilities, offering data support for ecological responsibility. This strengthens local governments' supervision of resources and the environment, promoting resource management in each region and contributing to the overall construction of ecological civilization.

Overcoming information difficulties is pivotal for high-quality decision-making, and the NRBS emerges as a valuable reference for local government resource management decision-making. Compiling the NRBS enhances the transparency and comparability of natural resource and environmental information, providing a standardized framework that facilitates horizontal and vertical comparisons among different regions and sectors. This aids governments at all levels in assessing their resource management situations, promptly identifying issues, and formulating more rational policies and measures for sustainable resource use and adequate environmental protection. The disclosure of detailed data and information during the compilation process also facilitates oversight of decisions and behaviors of subordinate departments, uncovering risks and potential problems in resource utilization and promoting compliance.

Based on the above analysis, we propose hypothesis 1.

H1 Compiling the natural resources balance sheet can significantly improve the level of local ecological civilization construction.

#### 3.2.2 The mechanism between the natural resources balance sheet and ecological civilization construction

The positive impact of government transparency on governance efficiency in the information age has been widely acknowledged [18,19]. Generally, Transparency is recognized as a critical factor affecting efficiency, and the higher the transparency, the more information is available, enhancing the efficiency of municipal authorities in managing scarce public administrative resources effectively [20].Within China's environmental governance system, the government plays a crucial role, particularly at the local level. Local governments, as primary drivers of local ecological civilization construction, have the responsibility to regulate natural resource usage, alleviate resource pressure, and strive for a harmonious balance between the environment and economic development [21].

The NRBS is a critical tool in mitigating information asymmetry between central and local governments. It provides essential information support for the performance assessment of the central government, thereby increasing institutional pressure on local governments. Operating within the "institutional pressure-organizational response" mechanism, local governments are compelled to implement practical measures in policy planning, financial support, and human resources investment to meet performance assessment requirements and address public environmental demands. Consequently, this enhances the governance efficiency of local government environmental management. Simultaneously, the NRBS offers comprehensive local government resource and environmental management information. This enhances the accuracy and efficiency of decision-making, propelling the progress of local ecological civilization construction.

Based on the above analysis, we propose hypothesis 2:

The natural resources balance sheet affects ecological civilization construction through environmental governance efficiency.

**Fig. 1:**
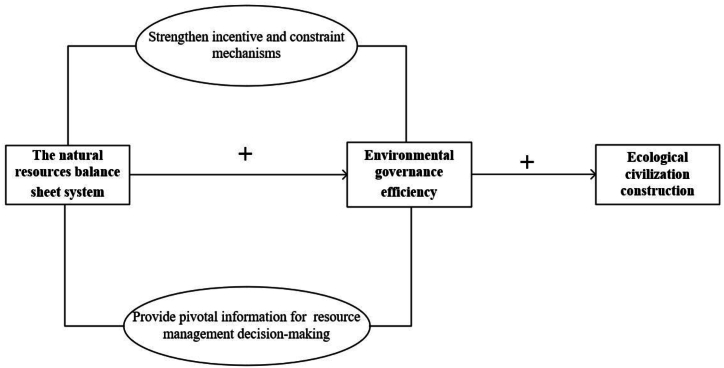
Theoretical framework.

The theoretical framework of our paper is illustrated in Fig. 1.

## 4 Research design

### 4.1 Sample and data

Our study centers on the impact of compiling the NRBS on the local ecological civilization construction. According to the pilot program, in 2015, Hulunbuir in the Inner Mongolia Autonomous Region, Huzhou in Zhejiang Province, Loudi in Hunan Province, Chishui in Guizhou Province, and Yan'an in Shanxi Province actively initiated the pilot work of compiling NRBS. Chengde in Hebei Province also voluntarily conducted the trial compilation of NRBS. As our paper primarily employs data from prefecture cities, and Chishui City in Guizhou Province is a county within the jurisdiction of a prefecture city, its data is excluded to enhance the accuracy of estimation results. Therefore, we consider the cities that conducted the compilation of NRBS in 2015 as the treatment group, while the cities in the same provinces that did not undergo the compilation serve as the control group.

According to the NRBS work plan, the trial compilation of NRBS commenced in all provinces nationwide from 2018 onward. To eliminate interference from other cities, experimental data after 2018 would not be considered. Primary data sources are the "China Regional Statistical Yearbook" and "China Urban Statistical Yearbook" from 2012 to 2018, provincial statistical yearbooks, and annual budget reports of various cities. Missing data are manually supplemented based on statistical bulletins from each prefecture city. We apply the logarithmic transformation to some control variables in the regression model to reduce the absolute differences.

### 4.2 Variable measurement

#### 4.2.1 Level of ecological civilization Construction(ECCL)

In order to assess the level of local ecological civilization construction, we refer to the existing research results and the Chinese government's Ecological Civilization Construction Appraisal Goal System. We construct a set of indicator systems for measuring the ecological civilization level of cities that contains four significant themes: ecological nature, ecological economy, ecological society, and environmental governance. We adopted the entropy weight method topsis evaluation model to evaluate each city's ecological civilization construction level. Details of the ECCL index system can be found in Appendix Table A1.

#### 4.2.2 The NRBS system

We use whether the city compiles the NRBS as a dummy variable for policy grouping (TREAT), which takes the value of 1 for areas that compile NRBS and 0 otherwise. We use the start of the pilot NRBS program as a dummy variable for temporal grouping (POST), in which we take the value of 0 before 2015 and 1 for 2015 and after that. The interaction term of TREAT and POST(TREAT*POST) is the core explanatory variable. The interaction term coefficient (TREAT*POST) measures the difference in policy effects. If the coefficient of TREAT*POST is significantly positive, it indicates that compiling the NRBS can effectively improve the level of local ecological civilization.

#### 4.2.3 Environmental governance efficiency (EGE)

The Data Envelopment Analysis (DEA) has become a widely adopted tool for efficiency testing, particularly in the fields of energy and the environment [22]. Drawing on insights from Peng et al.’s research [23], we employ the DEA-SBM model to measure environmental governance efficiency of the local governments. We carefully select input and output indicators to ensure a comprehensive evaluation. The chosen input indicators include government environmental protection expenditure and the number of environmental management practitioners. Meanwhile, the desired output indicators encompass the comprehensive utilization rate of industrial solid waste and the green space area. Additionally, we consider non-desired output indicators, such as tons of industrial wastewater emissions, industrial smoke (powder) dust emissions, and industrial sulfur dioxide emissions.

#### 4.2.4 Control variables

In order to accurately screen the effectiveness of the NRBS system, the measurement model introduces the indicators of economic development level, industrialization degree, foreign direct investment, population density, and infrastructure level as control variables. The economic development level (EDL) is expressed as the logarithm of the regional GDP of each prefecture-level city; the degree of industrialization (INDDEG) is expressed as the ratio of the value added of the secondary industry to the total regional economic output of each prefecture-level city; foreign direct investment (FDI) is expressed as the amount of the actual use of foreign capital in the current year; Population density (PD) is expressed using the number of people per square kilometer of land in the region; and the level of infrastructure (RA) is measured by road area per capita. Table A2 in the appendix shows the definitions of variables.

### 4.3 Model specification

The Difference-in-Differences (DID) model is one of the most popular methods in causal inference and policy effects analysis. By contrasting the impacts of exogenous shocks on treatment and control groups, the DID model provides a robust framework for discerning causal relationships. In this study, we take the NRBS system as an exogenous shock and construct a quasi-natural experiment to analyse the impact of the NRBS system by comparing the difference in the changes of ecological civilization construction in the areas of the treatment group and the control group before and after the NBRS pilot system. Referring to Zhou et al. (2024) [24],we construct the research model as follows:

(1) $${\text{ECCL}}_{\text{it}}=\alpha +\delta {\text{TREAT}}_{i}\times {\text{POST}}_{t}+\mu_{i}+\lambda_{t}+\eta X_{\text{it}}+\varepsilon_{\text{it}}$$

Where μ_i_ is the individual fixed effect, λ_t_ is the time fixed effect, X_it_ is the other control variables, and the interaction term TREAT_i_ × POST_t_ represents the actual effect of the treatment group in the treatment period, whose coefficients are precisely the treatment effects we are interested.

We use the stepwise method proposed by Baron and Kenny (1986) to test the mediation effect. Widely embraced in social science research, this method systematically evaluates how independent variables influence dependent variables through intermediate variables. The stepwise approach sequentially assesses the significance of direct and indirect effects, shedding light on the mediating role of the intermediate variable. The mediation effect test model in our study is set up as follows:

The mediation effect test model is set up as follows:

(2) $${\text{EGE}}_{\text{it}}=\alpha +\delta {\text{TREAT}}_{i}\times {\text{POST}}_{t}+\mu_{i}+\lambda_{t}+\eta X_{\text{it}}+\varepsilon_{\text{it}}$$

(3) $${\text{ECCL}}_{\text{it}}=\alpha +\delta {\text{TREAT}}_{i}\times {\text{POST}}_{t}+\beta {\text{ECG}}_{\text{it}}+\mu_{i}+\lambda_{t}+\eta X_{\text{it}}+\varepsilon_{\text{it}}$$

## 5 Empirical results

### 5.1 Descriptive statistics and discussions

The descriptive statistics of the main variables are presented in Table 1. The ecological civilization construction level (ECCL) exhibits significant variability, with a maximum value of 0.515, a minimum of 0.063, and an average of 0.145, indicating substantial diversity among prefectural municipalities. The economic development level (EDL) is between 9.511 (maximum) and 5.610 (minimum), with a standard deviation of 0.757, suggesting a relatively homogeneous level of economic development across the prefectural cities. The industrialization degree (INDDEG) ranges from a maximum of 67.340 to a minimum of 26.510, with a mean of 47.734, indicating considerable disparity in industrialization levels across prefectural cities. Other control variables fall within reasonable ranges, suggesting no outliers or extreme observations. The slight difference between the mean and median of variables implies a symmetrical distribution, aligning with the assumption of approximate normal distribution.

**Table 1:** Descriptive statistics of variables.

Variables	N	Mean	Std. Dev.	Median	max	min
ECCL	378	0.145	0.077	0.116	0.515	0.063
TREAT	378	0.093	0.290	0	1	0
POST	378	0.571	0.496	1	1	0
EGE	378	0.316	0.272	0.219	1	0.009
EDL	378	7.583	0.757	7.495	9.511	5.610
INDDEG	378	47.734	8.915	48.100	67.340	26.510
FDI	378	12.069	1.725	12.298	15.382	6.464
PD	378	8.062	0.711	8.114	9.397	5.740
RA	378	2.502	0.564	2.603	4.054	0.385

### 5.2 Basic regression results

The net effect of the NRBS system on local ecological civilization construction is estimated using the baseline regression model (1). Table 2, Column (1), presents regression results controlling for individual and time-fixed effects. The coefficients of the NRBS system on ecological civilization construction are significantly positive (δ1 = 0.014, p < 0.05), affirming that the preparation of NRBS significantly improves urban ecological civilization construction, validating Hypothesis 1. With the addition of control variables (Column 2), the net impact coefficient remains significant at the 5 % level, robustly supporting the conclusion that the natural resources balance sheet significantly promotes local ecological civilization construction. Our findings bolster the perspective of Bringezu et al. (2016), who underscore the indispensable nature of information regarding natural resource utilization for the consistent implementation of sustainable development goals, and demonstrate how natural resource use accounts can inform these goals [25].

**Table 2:** DID model test results.

Variables	(1)	(2)
	ECCL	ECCL
TREAT* POST	0.014**	0.015**
	(2.11)	(2.21)
PD		−0.006**
		(-1.99)
RA		−0.003
		(-0.82)
EDL		0.038***
		(3.22)
INDDEG		−0.001***
		(-3.62)
FDI		0.002
		(1.21)
Constant	0.127***	−0.048
	(49.99)	(-0.56)
City FE	Yes	Yes
Year Effect	Yes	Yes
Observations	378	378
R-squared	0.353	0.402

Note: ***, **, and * indicate p < 0.01, p < 0.05, and p < 0.1, respectively.

### 5.3 Robustness test

#### 5.3.1 Parallel trend test

**Fig. 2:**
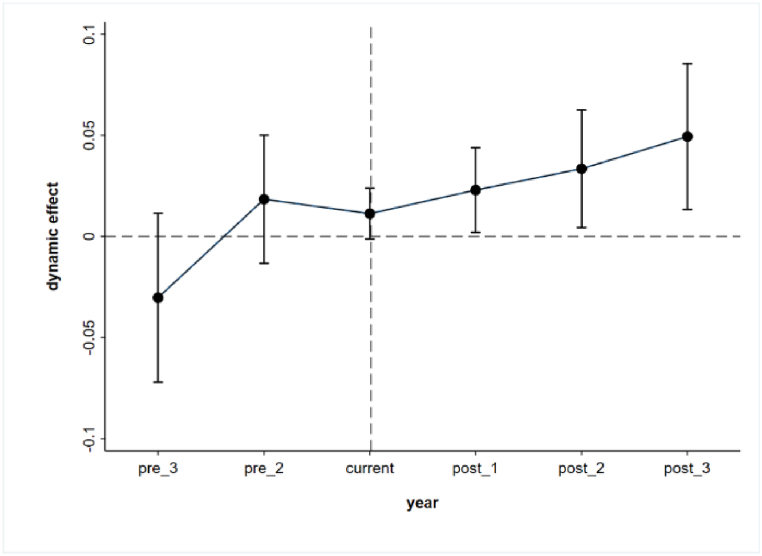
Parallel trend graph.

**Fig. 3:**
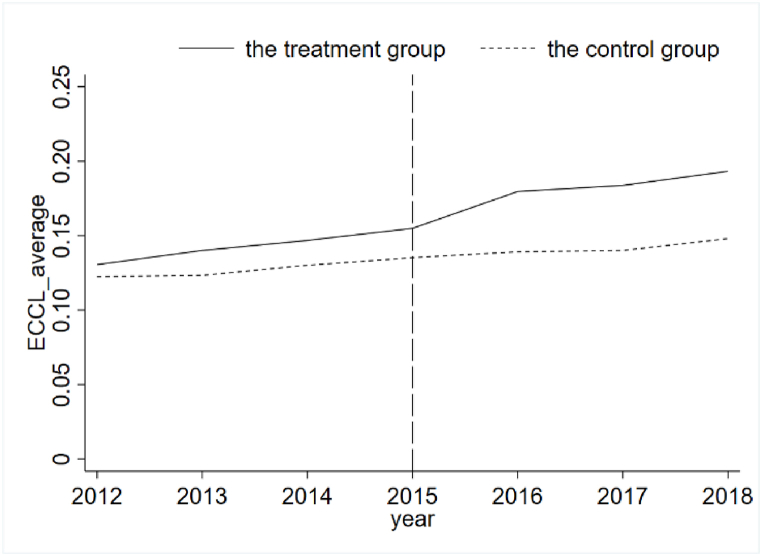
Trends in the level of ecological civilization in prefecture-level cities.

A parallel trend test is conducted using the event study method proposed by Jacobson et al. (1993) to meet the parallel trend assumption. As shown in Fig. 2, none of the TREAT* POST coefficients are significant until 2015, indicating that before the implementation of the NRBS system, there is no significant difference in the level change of ecological civilization construction between the treatment group of cities and the control residence localities, which satisfies the assumption of parallel trend. Meanwhile, we present a graph of the average annual change in the trend of ecological civilization construction in localities after 2015, as shown in Fig. 3. It shows that after the implementation of the policy, the level of ecological civilization construction in the treatment group of cities is significantly increased relative to the control group of cities.

#### 5.3.2 Placebo testx

**Fig. 4:**
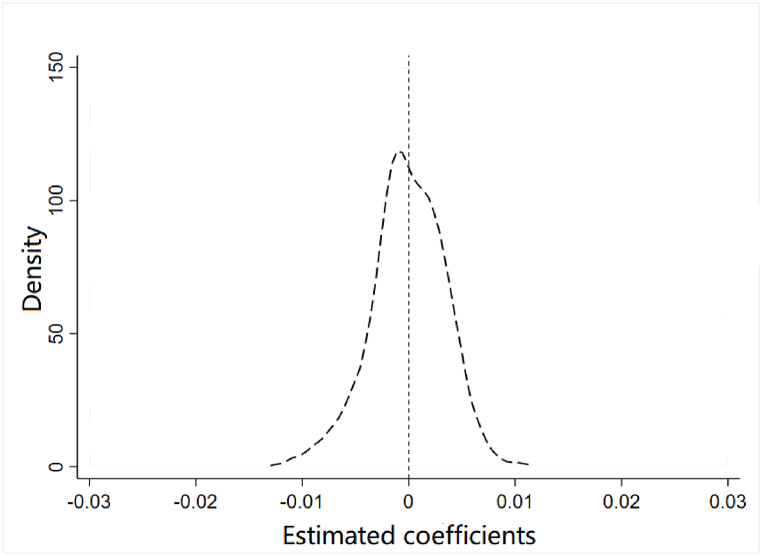
Placebo test.

We chose the following two placebo tests. Counterfactual Test: Setting the policy implementation time to 2014, the non-significant coefficient of TREAT*POST (Table 4, Column 1) confirms the robustness of the baseline results. Randomized generation of experimental groups: The placebo test (Fig. 4) demonstrates a normal distribution, suggesting a small probability event, and essentially excludes the influence of unknown factors on the regression results.

#### 5.3.3 Other robustness tests

We conduct three further robustness tests for our research finding. First, we employ PSM to match control groups and conduct regression with the matched data (Table 3, Column 2), yielding significantly positive TREAT*POST coefficients consistent with baseline results. Second, we exclude the effects of other policies: controlling for the central environmental protection inspection system. the TREAT*POST coefficients in the regression (Table 5, Column 3) maintain significance and direction, confirming the robustness of the findings. Third, we choose to replace the regression model and lag policy treatment time: The mixed regression model and lagged policy treatment time (Table 3, Columns 4 and 5) produce significantly positive coefficients for TREAT*POST, supporting the robustness of the benchmark results.

**Table 3:** Robustness test results.

Variables	(1)	(2)	(3)	(4)	(5)
	ECCL	ECCL	ECCL	ECCL	ECCL
TREAT* POST	0.011	0.016**	0.014**	0.051***	0.016**
	(0.76)	(2.53)	(2.18)	(4.52)	(2.36)
PD	−0.006**	0.001	−0.006*	−0.012***	−0.006**
	(-2.01)	(0.22)	(-1.96)	(-3.02)	(-1.98)
RA	−0.003	−0.001	−0.004	−0.001	−0.004
	(-1.01)	(-0.31)	(-0.88)	(-0.15)	(-0.95)
EDL	0.037*	0.024*	0.035***	0.041***	0.038***
	(1.69)	(1.89)	(2.85)	(7.88)	(3.19)
INDDEG	−0.001***	−0.001***	−0.001***	−0.001***	−0.001***
	(-3.00)	(-2.89)	(-3.58)	(-4.34)	(-3.56)
FDI	0.002*	0.002	0.002	0.007***	0.002
	(1.68)	(1.15)	(1.22)	(2.74)	(1.26)
SUPERVISION			0.003		
			(0.97)		
Constant	−0.042	−0.022	−0.026	−0.044	−0.045
	(-0.28)	(-0.25)	(-0.29)	(-0.93)	(-0.53)
City FE	Yes	Yes	Yes	Yes	Yes
Year Effect	Yes	Yes	Yes	Yes	Yes
Observations	378	338	378	378	378
R-squared	0.397	0.364	0.404	0.638	0.403

**Table 4:** Mediated effects of local government environmental governance efficiency: Regression results.

Variables	(1)	(2)
	EGE	ECCL
TREAT* POST	0.065*	0.014**
	(1.96)	(2.22)
ECE		0.021***
		(3.03)
Controls	Yes	Yes
Constant	0.143	−0.051
	(0.21)	(-0.60)
City FE	Yes	Yes
Year Effect	Yes	Yes
Observations	378	378
R-squared	0.095	0.419

**Table 5:** Moderating effect test results.

Variables	(1)	(2)
	ECCL	ECCL
TREAT* POST	0.006*	0.056***
	(1.99)	(4.34)
TREAT* POST*AUDIT	0.009***	
	(2.86)	
TREAT* POST*PEC		0.001***
		(3.17)
Controls	Yes	Yes
Constant	(1.14)	−0.074
	−0.055	(-0.92)
City FE	Yes	Yes
Year Effect	Yes	Yes
Observations	378	378
R-squared	0.405	0.490

### 5.4 Mechanism test results

Table 4 presents the results of the mediated effects model test. In Column (1) of Table 4, when the independent variable is local government environmental governance efficiency, the coefficient of TREAT*POST is significantly positive at the 10 % level. Implementing the natural resource asset compilation system positively influences local government environmental governance efficiency, aligning with theoretical expectations. In Column (2) of Table 4, the coefficients of TREAT*POST and ECE are 0.014 and 0.021, respectively. Both coefficients are statistically significant at the 1 % and 5 % levels, indicating the presence of a mediating effect of local government environmental governance efficiency between the NRBS and local ecological civilization construction. This finding supports hypothesis 2. These regression results underscore the pivotal role of local government environmental governance efficiency as a mediating factor, shedding light on the pathway through which the NRBS influences the construction of local ecological civilization. Our empirical results align with the findings of Zhao et al. (2023). They suggest that when regional information transparency is low, the public disclosure of regional environmental information can effectively enhance natural resource use efficiency, which is crucial for achieving green recovery and sustainable development [26].

## 6 Additional analysis

### 6.1 Moderating effect: the outgoing audit of natural resources

As previously emphasized, the NRBS, containing rich resources and environmental information, plays a pivotal role in promoting ecological civilization construction. Realizing its potential requires the central government to introduce corresponding supporting systems, with the outgoing audit of natural resources being integral to this process. This audit primarily scrutinizes the "ecological account" of leading cadres, serving as a crucial criterion for their assessment, appointment, dismissal, reward, and punishment, reinforcing the continued utilization of the NRBS. Additionally, the outgoing audit of natural resources facilitates a comprehensive assessment and review of the preparation of the NRBS, identifying areas for improvement. This synergy between the two systems forms a virtuous circle, allowing for the full realization of their combined effect. Consequently, the natural resource audit of leading cadres is posited to enhance information support for the NRBS and further the construction of local ecological civilization.

To examine the moderating effect of the outgoing audit of natural resources, this study introduces the dummy variable "AUDIT" and generates its cross-multiplier with the core explanatory variable (TREAT* POST). This interaction term, TREAT* POST*AUDIT, is then added to Model (1) for regression. The result is presented in in Column (1) of Table 5. The estimated coefficients of TREAT*POST*AUDIT are all significantly positive, signifying that the outgoing audit of natural resources positively regulates the relationship between the NRBS and local ecological civilization construction. In areas with audits, cities are better positioned to leverage the natural resources balance sheet to promote ecological civilization construction than those without outgoing audits.

### 6.2 Moderating effect: public concern for the environment

As direct perceivers of the ecological environment, the public exerts informal institutional pressure on the government through environmental concern and demands. Higher public concern for environmental issues implies more significant public opinion pressure and social responsibility for the government. The public's concern necessitates positive government responses, such as environmental information disclosure. The preparation and disclosure of an NRBS constitute a significant measure in addressing public concern about the government's efforts to build an ecological civilization. This quantitative tool not only provides the public with essential information on the type, quantity, distribution, and utilization of regional natural resources but also records changes in resource dynamics, showcasing the government's efforts and achievements in ecological environmental protection.

Consequently, increased public concern for the environment may lead local governments to pay closer attention to preparing NRBS and implement ecological civilization construction policies more rigorously. The study introduces the cross-multiplier term of TREAT* POST and public environmental concern (PEC). The regression results are presented in Column (2) of Table 5. The regression coefficient of TREAT* POST*PEC is 0.009 and significant at the 1 % level. This indicates that in areas with higher public environmental concern, the natural resources balance sheet has a more pronounced effect on ecological civilization construction, highlighting a more significant promotion effect.

## 7 Conclusions

We employ the DID method to investigate the impact of the NRBS system on the construction of local ecological civilization in Chinese prefecture-level cities from 2012 to 2018. The findings are as follows: First, the NRBS compilation significantly elevates the level of local ecological civilization construction. Second, the mechanism test reveals that the efficiency of local government environmental governance acts as a mediator between the NRBS and ecological civilization construction. In other words, the NRBS system realizes its effect on ecological civilization construction by improving the efficiency of local government environmental governance. Third, the analysis of moderating effects indicates that the outgoing audit of natural resources and public environmental concern positively mediate the relationship between the NRBS and local ecological civilization construction.

This study shows that the NRBS has a significant impact on sustainable development, which provides three policy insights as follows: First, the government needs to accelerate the formulation of unified standards and guidelines for NRBS compilation. Clarify normative requirements for data collection, measurement, and reporting to ensure consistency and comparability across different regions and sectors. Strengthen data quality management and monitoring and establish an effective mechanism for data validation and verification to ensure the credibility and reliability of NRBS data, providing a more reliable basis for policy formulation and decision-making. Second, promote information sharing and resource integration between national risk and statistical reporting and auditing departments, and give full play to the synergy between natural resource asset audits of leading cadres and natural resource balance sheet compilation. To achieve this goal, the government should establish an integrated information-sharing platform facilitating real-time data dissemination and interdepartmental collaboration. By leveraging shared insights and resources, authorities can enhance their grasp of natural resource asset dynamics, formulate effective resource management strategies, and foster sustainable resource utilization and conservation. Third, the disclosure of information on NRBS must be strengthened. Leveraging the internet and new media technologies to establish comprehensive disclosure platforms for natural resource assets enhances public access to environmental data. Concurrently, furnishing detailed resource and environmental data reports, statistical analyses, and policy interpretations cultivates public awareness and engagement in ecological and environmental affairs. These initiatives promote the construction of a diversified governance model, which means that the public, government and business stakeholders can jointly contribute to promoting ecological civilization.

The study has some limitations that provide valuable insights for future research. Firstly, constrained by data availability, our analysis focuses solely on the immediate policy ramifications of the NRBS system, thus precluding an exhaustive examination of its long-term effects or their sustainability. Subsequent investigations could pivot from this standpoint to conduct a more expansive inquiry into the enduring policy impacts and sustainability. Secondly, our study overlooks the nuanced characteristics inherent to individual regions, such as the influence of contextual variables like governmental promotion pressures, local fiscal constraints, and the degree of financial development. These regional idiosyncrasies may significantly influence on the policy outcomes of the NRBS, yet they still need to be explored. Thus, future research could enhance comprehension of the variegated and intricate nature of policy outcomes across regions by incorporating these regional peculiarities. Finally, while the relevant department endeavor to ensure precision in crafting the NRBS, this does not guarantee comprehensive information quality assurance. The question of information quality assurance remains a salient concern, meriting further exploration in future studies.

## Ethical approval

This article does not contain any studies with human participants performed by any of the authors.

## Consent to participate

This article does not contain any studies with human participants performed by any of the authors.

## Data availability statement

Data will be available on request.

## CRediT authorship contribution statement

**Le Zhu:** Writing – review & editing, Writing – original draft, Visualization, Supervision, Software, Methodology, Formal analysis, Data curation, Conceptualization. **Yichuan Wang:** Writing – review & editing, Supervision, Conceptualization. **Shengchuan Guo:** Writing – review & editing, Validation, Data curation.

## Declaration of competing interest

The authors declare that they have no known competing financial interests or personal relationships that could have appeared to influence the work reported in this paper.

## Figures and Tables

**Appendix 1 Table A1:** Index System of ECCL

Theme	Index	Unit	Attribute	Weight
Eco-nature	Per capita water resources	Cubic meters/person	+	0.218
	Per capita cultivated land area	Hectares/person	+	0.240
	Percentage of forest cover	%	+	0.009
	Number of days with air quality at or better than level 2		+	0.011
Eco-economy	Per capita GDP	Yuan	+	0.082
	Tertiary sector as a percentage of GDP	%	+	0.031
	Public science and technology expenditures as a percentage of government expenditures	%	+	0.162
	Tertiary industry information transmission computer services and software employees	/	+	0.135
Eco-society	Total retail sales of consumer goods	Yuan	+	0.031
	Urbanization rate - %	%	+	0.017
	Public education expenditure as a percentage of government expenditure	%	+	0.034
	Per capita healthcare expenditure	Yuan	+	0.010
Environmental governance	Centralized treatment rate of sewage treatment plants	%	+	0.003
	Harmless treatment rate of domestic garbage	%	+	0.006
	Energy consumption per unit of GDP	Tons/yuan	–	0.006
	Sulfur dioxide emission intensity per unit of GDP	Kg/yuan	–	0.004

**Appendix 2 Table A2:** The Definitions of Variables

Variables	definitions
ECCL	Calculated and processed according to the evaluation index system, the greater the value of this variable, the higher the level of ecological civilization construction means
TREAT	Cities in the treatment group take 1, cities in the control group take 0
POST	0 until 2015, 1 for 2015 and beyond
EDL	the logarithm of the regional GDP of each prefecture-level city;
INDDEG	the ratio of the value added of the secondary industry to the total regional economic output of each prefecture city
FDI	the amount of the actual use of foreign capital in the current year
PD	the number of people per square kilometer of land in the region
RA	road area per capita.
